# Nanog Overcomes Reprogramming Barriers and Induces Pluripotency in Minimal Conditions

**DOI:** 10.1016/j.cub.2010.11.074

**Published:** 2011-01-11

**Authors:** Thorold W. Theunissen, Anouk L. van Oosten, Gonçalo Castelo-Branco, John Hall, Austin Smith, José C.R. Silva

**Affiliations:** 1 Wellcome Trust Centre for Stem Cell Research and Department of Biochemistry, University of Cambridge, Tennis Court Road, Cambridge CB2 1QR, UK; 2 Wellcome Trust/Cancer Research UK Gurdon Institute, University of Cambridge, Tennis Court Road, Cambridge CB2 1QN, UK

## Abstract

Induced pluripotency requires the expression of defined factors and culture conditions that support the self-renewal of embryonic stem (ES) cells [1]. Small molecule inhibition of MAP kinase (MEK) and glycogen synthase kinase 3 (GSK3) with LIF (2i/LIF) provides an optimal culture environment for mouse ES cells [2] and promotes transition to naive pluripotency in partially reprogrammed (pre-iPS) cells [3]. Here we show that 2i/LIF treatment in clonal lines of pre-iPS cells results in the activation of endogenous Nanog and rapid downregulation of retroviral Oct4 expression. Nanog enables somatic cell reprogramming in serum-free medium supplemented with LIF, a culture condition which does not support induced pluripotency or the self-renewal of ES cells, and is sufficient to reprogram epiblast-derived stem cells to naive pluripotency in serum-free medium alone. Nanog also enhances reprogramming in cooperation with kinase inhibition or 5-aza-cytidine, a small molecule inhibitor of DNA methylation. These results highlight the capacity of Nanog to overcome multiple barriers to reprogramming and reveal a synergy between Nanog and chemical inhibitors that promote reprogramming. We conclude that Nanog induces pluripotency in minimal conditions. This provides a strategy for imposing naive pluripotency in mammalian cells independently of species-specific culture requirements.

## Highlights

► Response to dual kinase inhibition (2i) is examined in clonal lines of pre-iPS cells ► Nanog enhances reprogramming in synergy with 2i or inhibition of DNA methylation ► Nanog counteracts p-Erk and high levels of Oct4 during somatic cell reprogramming ► Nanog is sufficient to reprogram epiblast-derived stem cells to naive pluripotency

## Results and Discussion

### Investigating the Response to Kinase Inhibition in Clonal Lines of Pre-iPS Cells

**Figure 1:**
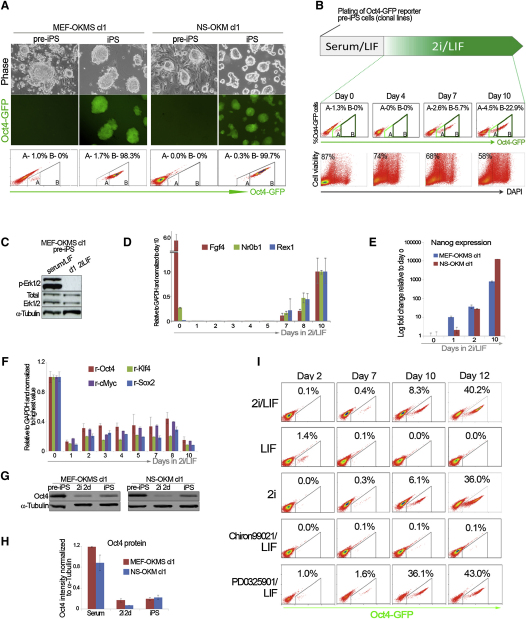
Characterization of the Response to Kinase Inhibition in Clonal Lines of Pre-iPS Cells (A) Top: phase and Oct4-GFP images of MEF-OKMS clone 1 and NS-OKM clone 1 pre-iPS cells cultured on a MEF feeder layer in serum/LIF conditions, and iPS cells derived in 2i/LIF from the same clonal lines. Bottom: flow cytometry analysis indicates the proportion of cells with Oct4-GFP reporter activity. OKMS and OKM refer to combinations of retroviral Oct4, Klf4, c-Myc, and Sox2 transgenes. (B) Experimental system for assessing transcriptional dynamics in clonal lines of pre-iPS cells during switch from serum/LIF to 2i/LIF conditions. Flow cytometry diagrams indicate the proportion of cells positive for the Oct4-GFP reporter transgene, and the proportion of live cells at daily time points during 2i/LIF treatment of pre-iPS cells (MEF-OKMS clone 1). Inlaid percentages in cell viability charts indicate the proportion of DAPI-negative (live) cells at each time point. (C) Western blot analysis for p-Erk1/2 and total Erk1/2 protein expression in pre-iPS cells cultured for 1 day in 2i/LIF medium. (D) Time course qRT-PCR analysis of endogenous pluripotency genes Fgf4, Nr0b1, and Rex1 during switch from serum/LIF (d0) to 2i/LIF conditions in pre-iPS cells. Error bars indicate the range of fold change relative to the day 10 sample. (E) Time course qRT-PCR analysis of Nanog expression after switching pre-iPS cells from serum/LIF to 2i/LIF conditions. Relative expression is shown on a logarithmic scale. Error bars indicate the range of fold change relative to the day 0 sample. (F) Time course qRT-PCR analysis of retroviral transgene expression during switch from serum/LIF (d0) to 2i/LIF conditions in pre-iPS cells. Error bars indicate the range of fold change relative to the day 0 sample. (G) Western blot analysis for Oct4 protein expression in pre-iPS cells cultured in serum/LIF or for 2 days in 2i/LIF and 2i-iPS cells. (H) Infra-red quantification of Oct4 protein intensity relative to α-tubulin in the samples shown in (G). Error bars indicate SD from analysis of two gels. (I) Time course flow cytometry analysis of changes in Oct4-GFP reporter activity during switch of pre-iPS cells plated in serum/LIF to serum-free 2i/LIF or serum-free medium supplemented with LIF, 2i, the GSK3 inhibitor (CHIR99021) and LIF or the MEK inhibitor (PD0325901) and LIF. See also Figure S1.

Pre-iPS cells have successfully acquired a proliferative capacity but have not yet attained the transcriptional and epigenetic hallmarks of naive pluripotency [3–5].To establish clonal lines of pre-iPS cells, we first infected mouse embryonic fibroblasts (MEFs) and neural stem (NS) cells with retroviral transgenes. We then picked and expanded individual pre-iPS cell colonies in serum/LIF conditions. Transfer and passaging in serum-free 2i/LIF medium generated a culture of iPS cells with uniform Oct4-GFP reporter activity (Figure 1A) and the capacity to contribute to adult mice (see Figure S1A available online). Weak activity of the Oct4 reporter was detected in <2% of pre-iPS cells in serum/LIF conditions (Figure 1A). Individual GFP events in pre-iPS cells, however, were significantly less intense than in iPS cells obtained from the same clones in 2i/LIF. To clarify the identity of the subset of pre-iPS cells with weak Oct4-GFP reporter activity, we performed serial purification of GFP-positive pre-iPS cells to obtain sufficient amounts of pure material for transcriptional and epigenetic characterization (Figure S1B). Retroviral transgene expression was maintained in GFP-positive pre-iPS cells, but fully silenced in 2i-iPS cells derived from the same clonal lines (Figure S1C). GFP-positive pre-iPS cells expressed Fgf4 and Nr0b1, which are recurrently detected in partially reprogrammed cells [3, 4]. However, other markers of authentic pluripotency such as Nanog and Rex1 remained undetectable in these cells. The *Nanog* promoter region was methylated in a pure sample of GFP-positive pre-iPS cells, but completely demethylated in 2i-iPS cells (Figure S1D). These results demonstrate that weak Oct4-GFP activity in clonal lines of pre-iPS cells in serum/LIF is not a sign of complete reprogramming. Consequently, 2i treatment does not select for expansion of an already resident pluripotent subpopulation, but actively induces conversion to pluripotency in pre-iPS cells.

To investigate the transcriptional response to 2i/LIF treatment in pre-iPS cells, we plated pre-iPS cells at clonal density on a feeder layer in serum/LIF until emergence of macroscopic colonies, at which point medium was switched to 2i/LIF. Oct4-GFP reporter activity was monitored at daily time points and samples were collected for gene expression analysis. Surprisingly, weak Oct4-GFP reporter activity initially disappeared completely upon switch to 2i/LIF (Figure 1B). This further indicates that sporadic Oct4-GFP reporter activity in serum/LIF reflects transient activation of the *Oct4* promoter and is not a sign of pluripotency (Figures S1C and S1D). By day 6 of 2i/LIF treatment, stable Oct4-GFP reporter activity began to appear in multiple colonies. The proportion of GFP-positive cells increased to approximately 30% by day 10. 2i treatment was accompanied by a degree of cell death, which became apparent 3 days after the medium switch (Figure 1B). Phospho-Erk (p-Erk) signal was completely extinguished within 24 hr of inhibitor treatment (Figure 1C). Fgf4 and Nr0b1 expression was initially downregulated upon switch to 2i/LIF, but reappeared during later time points together with other pluripotency markers (Figure 1D). In contrast, we observed a 30-fold upregulation of Nanog expression within 48 hr after switching to 2i/LIF (Figure 1E). Expression of Nanog increased steadily in subsequent time points. Unlike the initial reduction seen in pre-iPS cells, the same transcripts changed little in ES cells transferred from serum/LIF to 2i/LIF (Figures S1E–S1G). This confirms that the observed expression patterns are specific to pre-iPS cells as they reprogram.

In agreement with a recent study [6], we found that total expression of Oct4 and Klf4 was considerably higher in retrovirally derived pre-iPS cells than in ES cells (Figure S1H). However, we observed a reduction in retroviral transgene expression upon 2i/LIF treatment in pre-iPS cells (Figure 1F). Oct4 protein expression was 4 to 5-fold higher in pre-iPS cells compared with iPS cells and was significantly downregulated in pre-iPS cells 2 days after switching to 2i/LIF (Figures 1G and 1H; Figure S1I). This reduction in Oct4 expression was a combinatorial effect of the kinase inhibitors rather than serum depletion (Figures S1J–S1L). A modest increase in Oct4 expression is known to induce a differentiation program in ES cells [7]. We investigated the effect of further increases in Oct4 expression by stably transfecting a tamoxifen-inducible Oct4 vector in ES cells (Figure S1M) [8]. Induction of Oct4-ires-GFP occurred rapidly and by 48 hr ∼80% of cells were GFP-positive in two independent clones (Figure S1N). Oct4 overexpression induced an acute reduction in Nanog and Sox2 expression, which preceded modest induction of the differentiation markers Brachyury and Gata6 (Figures S1O and S1P). We infer that the levels of Oct4 observed in pre-iPS cells present an impediment to pluripotency gene expression.

We asked which component in 2i/LIF medium was responsible for the induction of pluripotency in pre-iPS cells. Switching to serum-free medium with LIF alone did not give rise to stable Oct4-GFP reporter activity (Figure 1I). Treatment with 2i in absence of LIF slowed the appearance of Oct4-GFP reporter activity compared with the 2i/LIF control induction, but reprogramming efficiency was still robust. A small number (0.1%) of strong Oct4-GFP events emerged after treating pre-iPS cells with the GSK3 inhibitor and LIF. Application of the MEK inhibitor and LIF, however, induced robust Oct4 reporter activity in up to 43% of cells by day 12. This was also the most selective culture medium we tested, with less than 40% cell viability after just 4 days of treatment. These data indicate that MEK inhibition is the main reprogramming cue in 2i and also exerts selection against pre-iPS cells.

### Nanog Enhances Reprogramming in Cooperation with 2i or Inhibition of DNA Methylation

**Figure 2:**
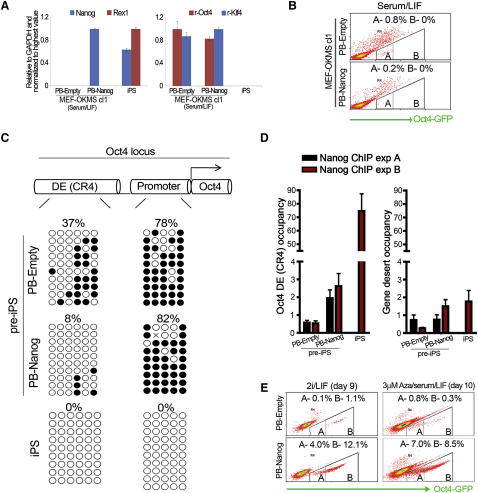
Nanog Enhances Reprogramming in Synergy with 2i or Inhibition of DNA Methylation (A) A *piggyBac* (PB) transgene was used to generate stable Nanog expressing cells in a clonal line of pre-iPS cells (MEF-OKMS clone 1). qRT-PCR analysis comparing expression of Nanog, Rex1, retroviral (r) Oct4 and r-Klf4 in PB-Nanog and PB-Empty pre-iPS cells expanded in serum/LIF. Error bars indicate the range of fold change relative to the sample with highest expression. (B) Flow cytometry analysis indicates the proportion of cells with Oct4-GFP reporter activity in both transgenic backgrounds in serum/LIF. (C) Bisulfite sequencing analysis of DNA methylation in the *Oct4* distal enhancer and promoter in PB-Empty and PB-Nanog pre-iPS cells and 2i-iPS cells. The percentage of methylated CpG sites is indicated above each methylation panel. (D) Chromatin immunoprecipitation analysis with a native Nanog antibody to the CR4 element in the *Oct4* distal enhancer and a gene desert control region. Results of two independent experiments are shown. iPS cell occupancy was not measured in experiment A. Occupancy is plotted as fold enrichment over IgG after normalization to the input, and error bars represent standard deviation of the technical replicates of the qPCR for each experiment. (E) Flow cytometry analysis comparing Oct4-GFP reporter activity after treatment of PB-Nanog and PB-Empty pre-iPS cells with 2i/LIF in serum-free medium or AZA (3 μM) in presence of serum/LIF. See also Figure S2.

Nanog was activated early in response to 2i/LIF treatment (Figure 1E) and occupies a central position in the transcriptional network regulating pluripotency [9, 10]. Using a loss-of-function approach we previously demonstrated that Nanog is necessary for the formation of embryonic and induced pluripotency [11]. In addition, Nanog was reported to accelerate reprogramming in a study using inducible lentiviral transgenes [12]. Here, we asked whether forced expression of Nanog might be sufficient to overcome the reprogramming block in pre-iPS cells. We made use of *PiggyBac* (PB) transposition [13] to introduce a transgene driving Nanog expression under control of a CAG promoter (PB-Nanog) in a clonal line of MEF-derived pre-iPS cells. Control transfectants expressing an empty vector transgene (PB-Empty) were generated in parallel. Expression of Nanog in stable transfectants expanded in serum/LIF was 1.5-fold higher compared with iPS cells. Forced expression of Nanog did not result in activation of the pluripotency marker Rex1 or downregulation of retroviral expression (Figure 2A). Lack of Oct4-GFP reporter activity further demonstrates that Nanog could not overcome the block to full reprogramming in presence of serum (Figure 2B). *Oct4* promoter methylation persisted in PB-Nanog pre-iPS cells (Figure 2C). In contrast, the percentage of methylated CpG sites in the *Oct4* distal enhancer was reduced from 38% in PB-Empty pre-iPS cells to 8% in PB-Nanog pre-iPS cells (Figure 2C). The distal enhancer is responsible for driving Oct4 expression in preimplantation embryos and ES cells [14] and contains the CR4 element, a critical binding site of Nanog in ES cells [15]. Chromatin immunoprecipitation analysis suggested that reduced CpG methylation might correlate with low level Nanog occupancy of the CR4 element in PB-Nanog pre-iPS cells (Figure 2D). However, Nanog occupancy was significantly higher in iPS cells.

Since the *Oct4* promoter remained hypermethylated in PB-Nanog pre-iPS cells, we asked whether chemical inhibition of DNA methylation could promote reprogramming to pluripotency. Indeed, treatment with the DNA methyltransferase inhibitor 5-aza-cytidine (AZA) for 10 days generated a significant proportion of cells with stable Oct4-GFP reporter activity in serum/LIF (Figure 2E). A small number of GFP-positive cells also emerged upon AZA treatment in PB-Empty or wild-type pre-iPS cells (Figure 2E; Figures S2A and S2B). Purification and subcloning of these GFP-positive cells generated a line of homogeneous iPS cells capable of contribution to chimeric mice (Figures S2C–S2E). This confirms a previous report that global inhibition of DNA methylation promotes direct reprogramming [4]. The de novo methyltransferase Dnmt3a was expressed in pre-iPS cells throughout 2i/LIF induction, while Dnmt3b expression was initially reduced and then upregulated (Figure S2F). Immunofluorescence analysis for Dnmt3b during 2i/LIF treatment showed weak cytoplasmic staining in pre-iPS cells and a stronger nuclear signal in iPS cells positive for both Oct4-GFP and Nanog protein (Figure S2G). This pattern shows resemblance to primordial germ cells where Dnmt3b is excluded from the nucleus at the time of DNA demethylation and epigenetic reprogramming [16]. The significant increase in efficiency of AZA-induced reprogramming in the PB-Nanog background reveals a synergy between Nanog and inhibition of global DNA methylation. We also examined reprogramming kinetics upon the application of 2i/LIF medium in PB-Nanog and PB-Empty pre-iPS cells. When transferred to 2i/LIF Oct4-GFP activity appeared earlier and the proportion of positive cells was more than 10-fold higher in the constitutive Nanog background (Figure 2E).

These results show that Nanog cooperates with distinct small molecules to enhance the efficiency of direct reprogramming. These pathways also intersect since endogenous Nanog was activated in response to 2i/LIF treatment in pre-iPS cells (Figure 1E), and suppression of p-Erk signaling results in increased Nanog expression in ES cells [17, 18]. Further gains in reprogramming yield may be obtained by integrating other chemical strategies that complement or reinforce the effects of these inhibitors. TGF-beta inhibition was reported to induce endogenous Nanog expression in partially reprogrammed cells but did not affect the kinase targets of the 2i cocktail [19]. Vitamin C treatment also promoted reprogramming in a MEK-independent manner and resulted in demethylation of the *Nanog* promoter [20]. This suggests that chemicals that promote or enhance the efficiency of reprogramming may converge on common transcriptional targets.

### Nanog Promotes Somatic Cell Reprogramming in Serum-Free Medium with LIF

**Figure 3:**
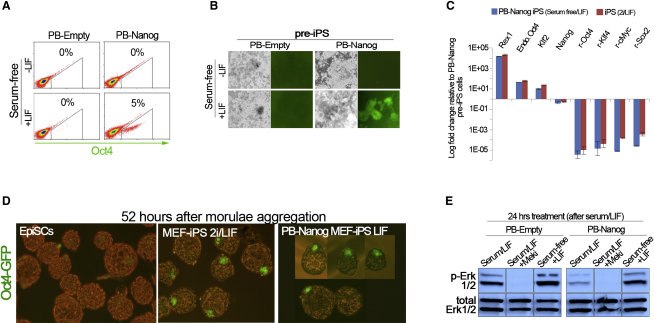
Nanog Promotes Somatic Cell Reprogramming in Minimal Conditions (A) Flow cytometry analysis of Oct4-GFP reporter activity in PB-Empty and PB-Nanog pre-iPS cells cultured for 2 weeks in serum-free medium or serum-free medium with LIF. (B) Phase and Oct4-GFP images of PB-Empty and PB-Nanog pre-iPS cells cultured for 2 weeks in serum-free medium or serum-free medium with LIF. (C) qRT-PCR analysis for endogenous pluripotency genes and retroviral transgenes in iPS cells derived from PB-Nanog pre-iPS cells in serum-free medium with LIF. Error bars indicate the range of fold change relative to PB-Nanog pre-iPS cells. (D) Oct4-GFP reporter activity in blastocysts 52 hr after morula aggregation of PB-Nanog iPS cells derived in serum-free medium with LIF. (E) Western blot analysis for p-Erk1/2 and total Erk1/2 protein expression in PB-Nanog and PB-Empty pre-iPS cells cultured for 1 day in serum/LIF with the MEK inhibitor PD0325901 or serum-free medium supplemented with LIF alone. See also Figure S3.

We then considered whether constitutive expression of Nanog might be sufficient to promote transition to pluripotency in serum-free conditions. No stable Oct4-GFP reporter activity was observed after switching PB-Nanog pre-iPS cells to serum-free medium alone. However, the Oct4-GFP reporter was robustly induced in presence of LIF (Figures 3A and 3B). PB-Empty pre-iPS cells did not give rise to stable GFP-expressing cells in either condition. GFP-positive cells that emerged in the constitutive Nanog background expressed Rex1, Klf2, and endogenous Oct4 and fully silenced retroviral transgenes (Figure 3C). To confirm this result in a different somatic origin, we introduced a PB-Nanog transgene in adult NS cells prior to retroviral infection (Figure S3A). Medium was switched after 5 days to serum-free medium with LIF or serum-free medium alone. Stable Oct4-GFP activity emerged in multiple colonies in serum-free medium with LIF within 7 days (Figure S3B). These GFP-positive cells had a pluripotent gene expression profile (Figure S3C). Since constitutive Nanog expression is likely to interfere with embryonic development, we opted to assess developmental potential 52 hr after morula aggregation, which corresponds to a late blastocyst stage. PB-Nanog iPS cells derived in serum-free medium with LIF readily colonized the epiblast and maintained homogeneous Oct4 reporter activity (Figure 3D; Figure S3D). In contrast, postimplantation epiblast-derived stem cells (EpiSCs) incorporated in the epiblast but Oct4-GFP reporter activity was greatly reduced 52 hr after aggregation.

These results demonstrate that constitutive expression of Nanog promotes transition to pluripotency in serum-free medium with LIF. The requirement of LIF for Nanog-induced reprogramming provides evidence that kinase inhibition has additional targets, since 2i was sufficient to convert pre-iPS cells to pluripotency in absence of LIF (Figure 1I). p-Erk levels were sustained in serum-free medium with LIF (Figure 3E). We also found that high Oct4 levels were unaffected after switching pre-iPS cells to serum-free medium with LIF (Figures S1J and S1K). Thus, Nanog can overcome the adverse effects of both p-Erk and high levels of Oct4 expression during reprogramming. We tested whether constitutive expression of Nanog can counteract the effects of Oct4 elevation in ES cells [7]. Expression of an episomal Oct4 transgene in ES cells caused pronounced differentiation by morphological criteria and the lack of alkaline phosphatase activity (Figures S3E and S3F). In contrast, self-renewal and expression of pluripotency genes were maintained upon coexpression of Oct4 and Nanog transgenes (Figures S3E–S3G). This indicates that Nanog safeguards establishment and maintenance of pluripotency against the effects of high levels of Oct4.

### Nanog Is Sufficient to Reprogram Epiblast-Derived Stem Cells to Naive Pluripotency

**Figure 4:**
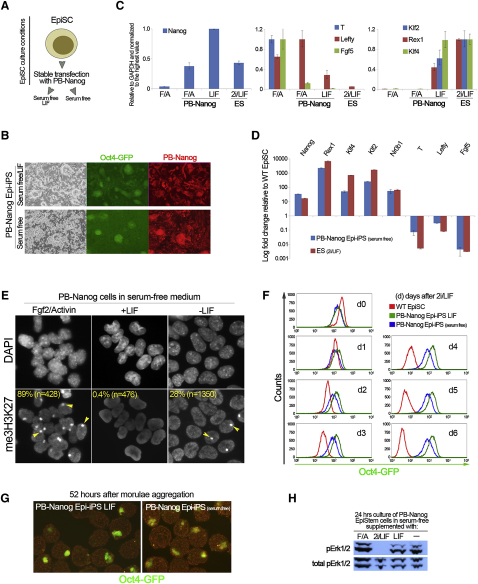
Nanog Is Sufficient to Reprogram Epiblast-Derived Stem Cells to Naive Pluripotency (A) Strategy for examining the sufficiency of Nanog in reprogramming of epiblast-derived stem cells (EpiSCs). A *piggyBac* (PB) Nanog-dsRed transgene was used to generate constitutive Nanog expressing EpiSCs, which also contain an Oct4-GFP reporter transgene. After selection in EpiSC culture conditions, PB-Nanog-dsRed EpiSCs were cultured in serum-free medium with LIF or serum-free medium alone. (B) Phase and Oct4-GFP images of emerging Epi-iPS colonies that were resistant to puromycin selection for the Oct4 reporter transgene. (C) qRT-PCR analysis of PB-Nanog EpiSCs in Fgf2 and Activin (F+A) and passage 1 PB-Nanog transfectants in serum-free medium with LIF. Error bars indicate the range of fold change relative to the sample with highest expression. (D) qRT-PCR analysis of PB-Nanog Epi-iPS cells derived in serum-free medium alone. Error bars indicate the range of fold change relative to wild-type EpiSCs. (E) Immunostaining for me3H3K27 of PB-Nanog EpiSCs cultured in Fgf2 and Activin, and PB-Nanog Epi-iPS cells derived in serum-free medium with LIF or serum-free medium alone. (F) Time course flow cytometry analysis of Oct4-GFP reporter activity after transferring wild-type (WT) EpiSCs and PB-Nanog Epi-iPS cells that were derived in serum-free medium or serum-free medium with LIF to 2i/LIF. (G) Fluorescence images showing Oct4-GFP reporter activity in blastocysts 52 hr after morula aggregation of PB-Nanog Epi-iPS cells derived in serum-free medium with LIF and serum-free medium alone. (H) Western blot analysis for p-Erk1/2 and total Erk1/2 protein expression in PB-Nanog-EpiSCs cultured in Fgf2 and Activin or for 1 day in 2i/LIF, LIF or serum-free medium alone.

Finally, we asked whether Nanog is sufficient to mediate reprogramming in absence of both exogenous self-renewing factors and other reprogramming transgenes. For this purpose we introduced a PB-Nanog transgene in EpiSCs, which can be reprogrammed to naive pluripotency by transfection with defined factors and culture in 2i/LIF [11, 13, 21]. Stable PB-Nanog transfectants were transferred to serum-free medium with or without LIF (Figure 4A). Under these conditions, PB-Nanog EpiSCs gave rise to putative iPS cell colonies (Epi-iPS) that were resistant to puromycin selection for the Oct4 reporter transgene in both conditions (Figure 4B). Establishment of naive pluripotency was confirmed by upregulation of ES-cell specific transcripts, and downregulation of EpiSC markers (Figures 4C and 4D). However, expression of Klf4 and Klf2 fell short of ES cell levels after transfer to serum-free medium alone. The silent X chromosome was epigenetically reprogrammed, but me3H3K27 foci were detected in 28% of cells after transfer to serum-free medium alone (Figure 4E). We then examined the response of PB-Nanog Epi-iPS cells to 2i treatment. This caused rapid downregulation of the Oct4-GFP reporter and progressive cell death in parental EpiSCs (Figure 4F). In contrast, PB-Nanog Epi-iPS cells obtained in both serum-free medium with LIF and serum-free medium alone maintained homogeneous Oct4-GFP reporter activity after 2i treatment (Figure 4F). To ascertain acquisition of bona fide pluripotency, we performed morula aggregations (Figure 4G). PB-Nanog Epi-iPS cells incorporated in the epiblast and maintained Oct4 reporter activity. We conclude that Nanog directs EpiSCs to naive pluripotency in conditions free of known exogenous self-renewing factors. p-Erk levels were sustained in these culture conditions (Figure 4H). The finding that LIF is dispensable for Nanog-induced reprogramming in EpiSCs but not somatic cells may reflect the fact that EpiSCs and ES cells already share expression of a significant number of pluripotency regulators [22, 23]. However, our data also indicate that LIF/STAT3 signaling stabilizes Epi-iPS cells transcriptionally and epigenetically. This is in agreement with the recent finding that activation of STAT3 is limiting during reprogramming of somatic cells and EpiSCs [24].

In this study, we investigated limiting components during the final stages of direct reprogramming. We found that Nanog has the capacity to overcome p-Erk signaling and high levels of Oct4 and enable reprogramming in minimal conditions. This result is significant in light of recent interest to generate a naive pluripotent state in cells of non-rodent origin, including human [25–27]. Conventional human ES cell culture conditions induce differentiation in naive pluripotent mouse cells [13]. By identifying factors such as Nanog that enable reprogramming in minimal conditions, it may be possible to bypass species-specific culture requirements and establish naive pluripotency in other mammalian species.

## Experimental Procedures

Pre-iPS cells were obtained by retroviral infection of NS cells or MEFs with pMXs-based retroviral reprogramming factors [1, 3]. Cultures were changed into ES cell medium (serum/LIF) at day 3 posttransduction. For further expansion, pre-iPS cells were replated onto feeders at day 5 in serum/LIF. Pre-iPS and NS cells were transfected using nucleofection (Amaxa) with 1 μg of PB-flox-Nanog-Pgk-Hygro plus 2 μg PBase expression vector, pCAGPBase [11]. For time course real-time PCR analysis of iPS cell induction, pre-iPS cells were plated in serum/LIF at clonal density on a STO or DsRed-expressing fibroblast feeder layer in 10 cm (3000 pre-iPS cells plated) or 6W (600 pre-iPS cells plated) format. Medium was switched to 2i/LIF when colonies reached macroscopic colony density after 6–8 days. Samples were collected daily for RNA extraction, either directly from whole pellets or after flow cytometric elimination of DsRed-expressing feeders. By time pre-iPS cell colonies had reached macroscopic density, feeders comprised no more than 2% of the complete culture. PB-Nanog iPS cells were at a proliferative disadvantage compared with pre-iPS cells in serum-free medium with LIF, requiring flow cytometric purification of cells positive for the Oct4-GFP reporter.

## Supplemental Information

Document S1. Three Figures, Supplemental Experimental Procedures, and One Table
