# Supplementary material for: Nanog Overcomes Reprogramming Barriers and Induces Pluripotency in Minimal Conditions
Source: Curr Biol. 2011 Jan 11;21(1):65–71. doi: 10.1016/j.cub.2010.11.074 (PMC3025321; doi:10.1016/j.cub.2010.11.074)
Supplement: Document S1. Three Figures, Supplemental Experimental Procedures, and One Table [file mmc1.pdf]

Current Biology, Volume *21*

Supplemental Information

Nanog Overcomes Reprogramming Barriers and Induces Pluripotency in Minimal Conditions

Thorold W. Theunissen, Anouk L. van Oosten, Gonçalo Castelo-Branco, John Hall, Austin Smith, and José C.R. Silva

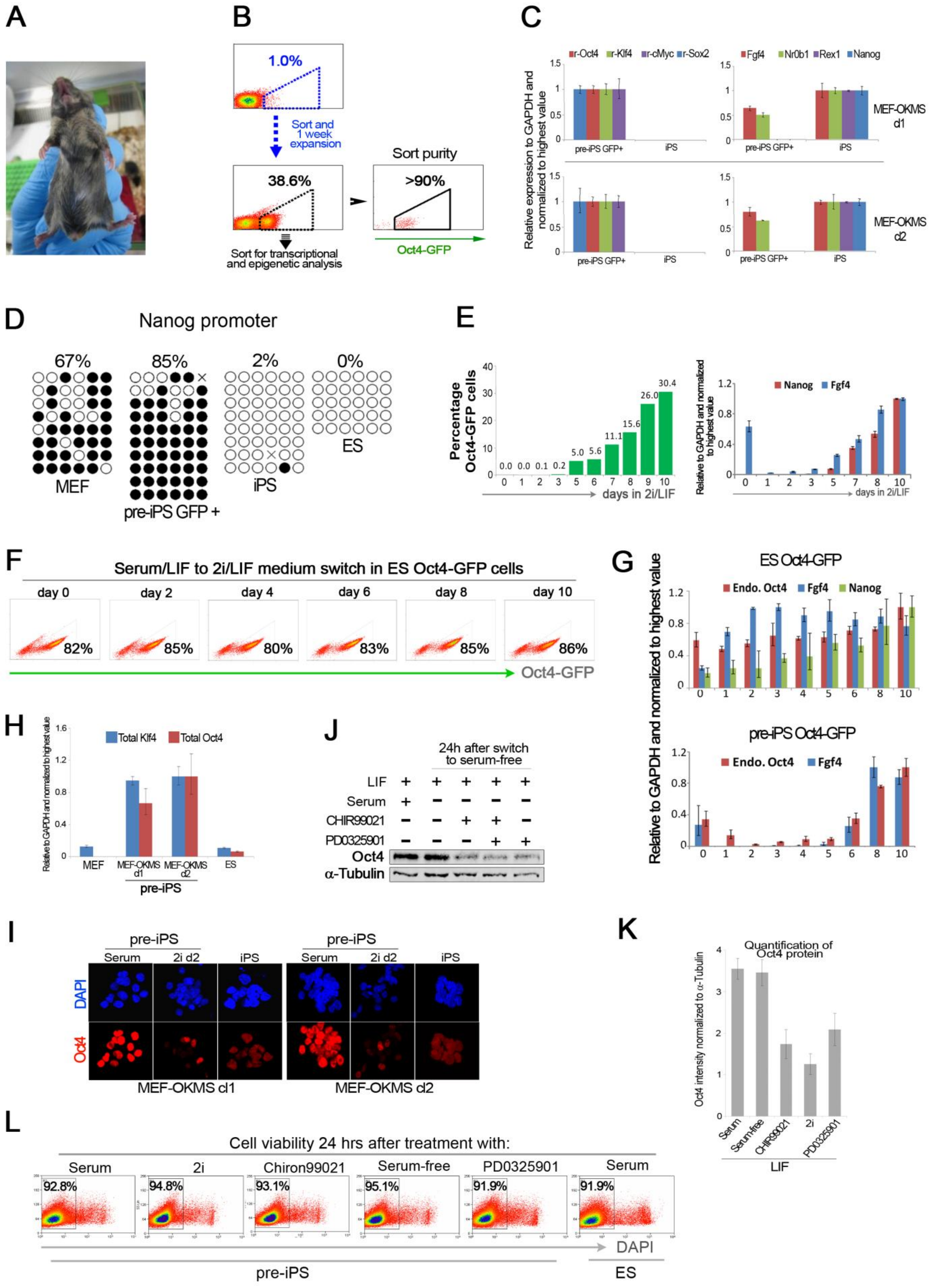
A
B
1.0%
Sort and 1 week expansion
38.6%
Sort purity
>90%
Sort for transcriptional and epigenetic analysis
Oct4-GFP
C
r-Oct4 r-Klf4 r-cMyc r-Sox2
Fgf4 Nr0b1 Rex1 Nanog
Relative expression to GAPDH and normalized to highest value
pre-iPS GFP+
iPS
MEF-OKMS cl1
MEF-OKMS cl2
D
Nanog promoter
67%
85%
2%
0%
MEF
pre-iPS GFP +
iPS
ES
E
Percentage Oct4-GFP cells
0.0 0.0 0.1 0.2 5.0 5.6 11.1 15.6 26.0 30.4
days in 2i/LIF
Relative to GAPDH and normalized to highest value
Nanog
Fgf4
F
Serum/LIF to 2i/LIF medium switch in ES Oct4-GFP cells
day 0
day 2
day 4
day 6
day 8
day 10
82%
85%
80%
83%
85%
86%
Oct4-GFP
G
ES Oct4-GFP
Endo. Oct4
Fgf4
Nanog
pre-iPS Oct4-GFP
Relative to GAPDH and normalized to highest value
H
Total Klf4
Total Oct4
Relative to GAPDH and normalized to highest value
MEF
MEF-OKMS cl1
MEF-OKMS cl2
ES
pre-iPS
J
24h after switch to serum-free
LIF
Serum
CHIR99021
PD0325901
Oct4
α-Tubulin
I
pre-iPS
Serum
2i d2
iPS
DAPI
Oct4
MEF-OKMS cl1
MEF-OKMS cl2
K
Quantification of Oct4 protein
Oct4 intensity normalized to α-Tubulin
Serum
Serum-free
CHIR99021
2i
PD0325901
LIF
L
Cell viability 24 hrs after treatment with:
Serum
2i
Chiron99021
Serum-free
PD0325901
Serum
92.8%
94.8%
93.1%
95.1%
91.9%
91.9%
DAPI
pre-iPS
ES

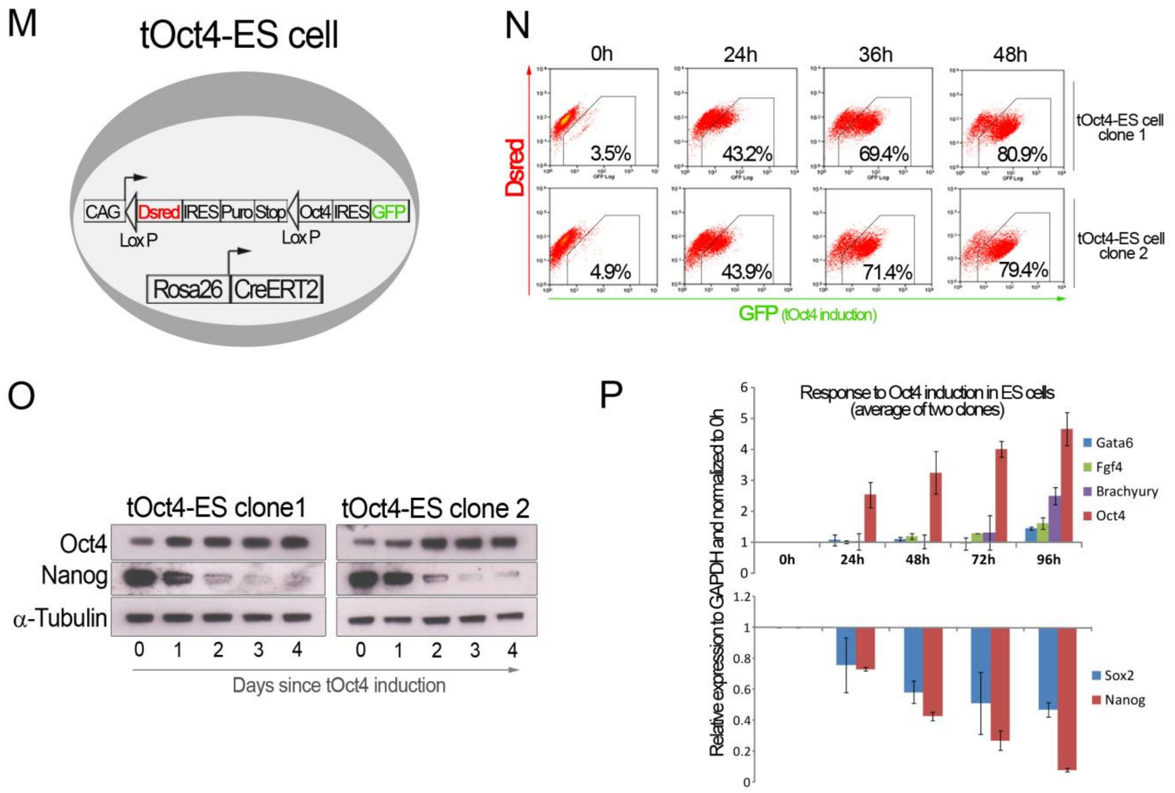

Figure S1. Characterization of the Response to Kinase Inhibition in Pre-iPS Cells (Related to Figure 1)

(A) Chimaeric mouse obtained after injecting 2i-iPS cells derived from NS-OKM pre-iPS clone 1 in C57/BL6 blastocysts. Agouti coat color indicates chimaeric contribution (MF1/129 background). Contribution to adult mice with germ-line transmission was previously shown for 2i-iPS cells derived from MEF-OKMS pre-iPS clone 1 [1].

(B) Serial flow cytometric purification for Oct4-GFP reporter activity in pre-iPS cells cultured in serum/LIF.

(C) Quantitative real time PCR (qRT-PCR) analysis of retroviral transgene and endogenous pluripotency gene expression in pre-iPS cells positive for the Oct4-GFP reporter. Error bars indicate the range of fold change relative to the sample with highest expression.

(D) Bisulfite sequencing analysis of DNA methylation in the *Nanog* promoter region in MEF-derived pre-iPS cells (cl1) positive for the Oct4-GFP reporter. The percentage of methylated CpG sites is indicated above each methylation panel.

(E) *(Left)* Quantification of Oct4-GFP reporter activity during 2i/LIF treatment of NS-OKM clone 1 pre-iPS cells, as measured by flow cytometry. *(Right)* Time-course qRT-PCR analysis of Fgf4 and Nanog expression during 2i/LIF treatment of NS-OKM clone 1 pre-iPS cells. Error bars indicate the range of fold change relative to the sample with highest expression.

(F) ES cells carrying an Oct4-GFP reporter transgene (ESO4G) were plated at clonal density on a MEF feeder layer in serum/LIF conditions. Once colonies reached macroscopic density (7 days), medium was switched to 2i/LIF and daily flow measurements were made of the proportion of cells with Oct4-GFP reporter activity.

(G) Time-course qRT-PCR analysis of Fgf4, endogenous Oct4 and Nanog expression in ES cells transferred from serum/LIF to 2i/LIF at clonal density. Error bars indicate indicate the range of fold change relative to the sample with highest expression.
(H) qRT-PCR analysis comparing expression of total Klf4 and Oct4 in MEF-derived pre-iPS cells in serum/LIF, MEFs and ES cells. Error bars indicate the range of fold change relative to the sample with highest expression.
(I) Immunofluorescence staining for Oct4 in pre-iPS cells cultured in serum/LIF or for 2 days in 2i/LIF, and 2i-iPS cells.
(J) Western blot analysis for Oct4 in pre-iPS cells (MEF-OKMS clone 1) plated in serum/LIF conditions and one day after switch to serum-free/LIF medium supplemented with either GSK3 inhibitor (CHIR99021), MEK inhibitor (PD0325901) or both (2i). This time point was chosen because it precedes the onset of cell death induced by either or both of the inhibitors (see Figure S1L).
(K) Infra-red quantification of Oct4 protein intensity relative to α-tubulin in the samples shown in (J). Error bars indicate SD from analysis of two gels.
(L) Flow cytometry diagrams indicate cell viability 24h after switching pre-iPS cells from serum/LIF to the conditions assessed for Western analysis. Inlaid percentages in cell viability charts indicate the proportion of DAPI-negative (live) cells in each condition.
(M) Schematic representation of ES cells carrying an inducible Oct4 transgene (tOct4-ES cell). A CreERT2 fusion protein is expressed from the Rosa26 locus.
(N) Treatment of tOct4 ES cells with 4-hydroxytamoxifen (4OHT) to induce Oct4 overexpression. Flow cytometry analysis monitoring the proportion of cells positive for Oct4-ires-GFP.
(O) Western blot analysis for Oct4 and Nanog protein during inducible Oct4 overexpression.
(P) Time-course qRT-PCR analysis of Oct4, Nanog, Fgf4, Brachyury, Gata6 and Sox2 expression during inducible Oct4 overexpression. Average fold change was calculated from two clonal inductions. Error bars indicate one SD.

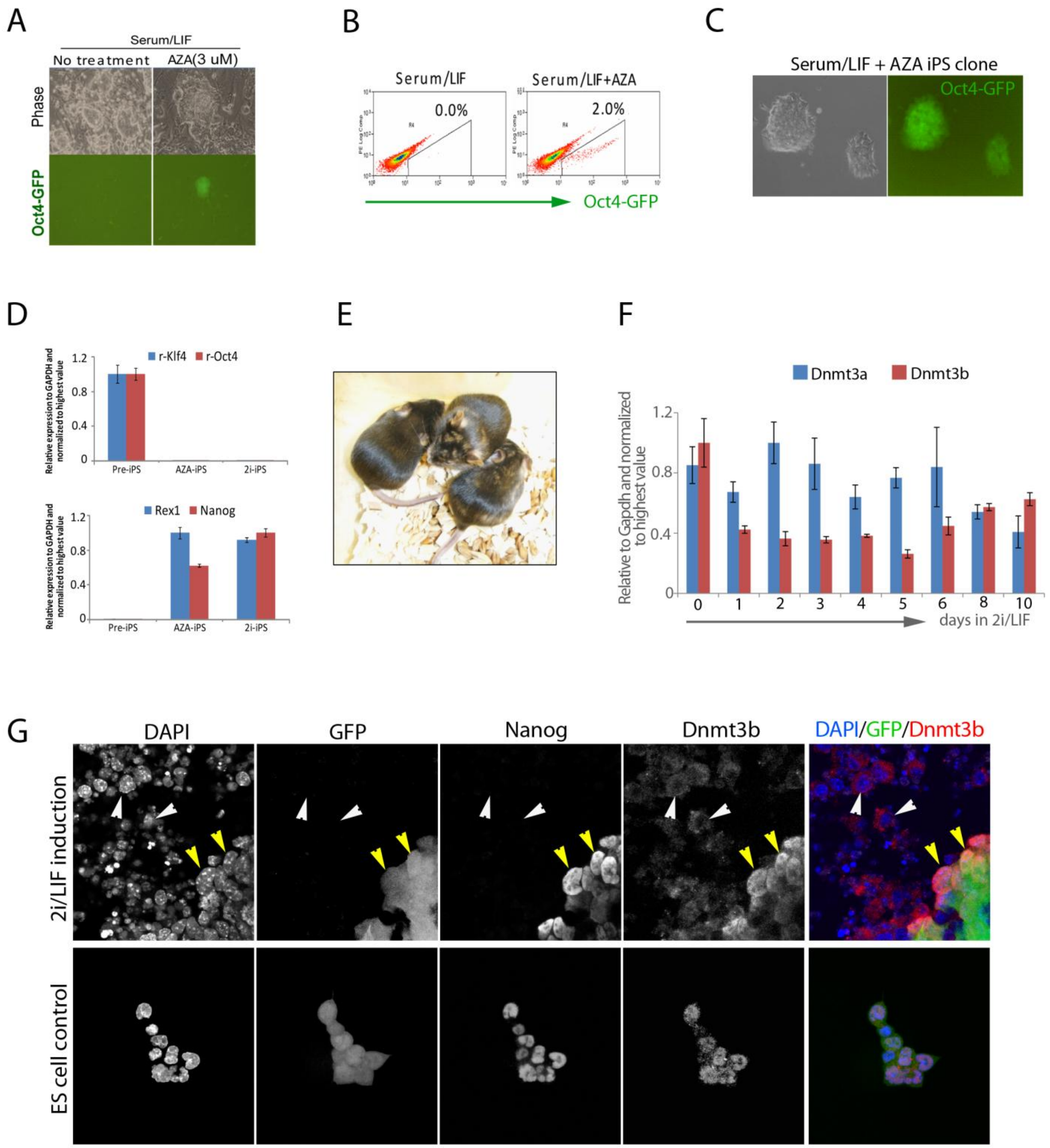

Figure S2. Inhibition of DNA Methylation Promotes Reprogramming to Pluripotency in Pre-iPS Cells (Related to Figure 2)

(A) Phase and GFP images obtained after culturing pre-iPS cells (MEF-OKMS clone 1) for 10 days in serum/LIF supplemented with 5-aza-cytidine (AZA) (3 μM).

(B) Flow cytometry analysis of Oct4-GFP reporter activity in pre-iPS cells after 10 days of treatment with AZA (3 μM).

(C) Phase and GFP images of a subclone of AZA-iPS cells derived from a clonal pre-iPS cell line (MEF-OKMS clone 1) by purifying the subset of GFP-positive cells that emerged in serum/LIF medium supplemented with AZA (3 μM).
(D) qRT-PCR analysis of retroviral Oct4, retroviral Klf4, Nanog and Rex1 expression in AZA-iPS cells compared to pre-iPS cells and 2i-iPS cells (MEF-OKMS clone 1). Error bars indicate the range of fold change relative to the sample with highest expression.
(E) Chimaeric mice obtained after injecting AZA-iPS cells derived from MF1/129 background in C57/BL6 blastocysts. Agouti coat color indicates chimaeric contribution.
(F) Time-course qRT-PCR analysis of Dnmt3a and Dnmt3b expression in pre-iPS cells (MEF-OKMS clone 1) transferred from serum/LIF to 2i/LIF at clonal density. Error bars indicate indicate the range of fold change relative to the sample with highest expression.
(G) Immunofluorescence staining for Dnmt3b in pre-iPS cells and emerging iPS cells after culture in 2i/LIF for 10 days and control ES cells.

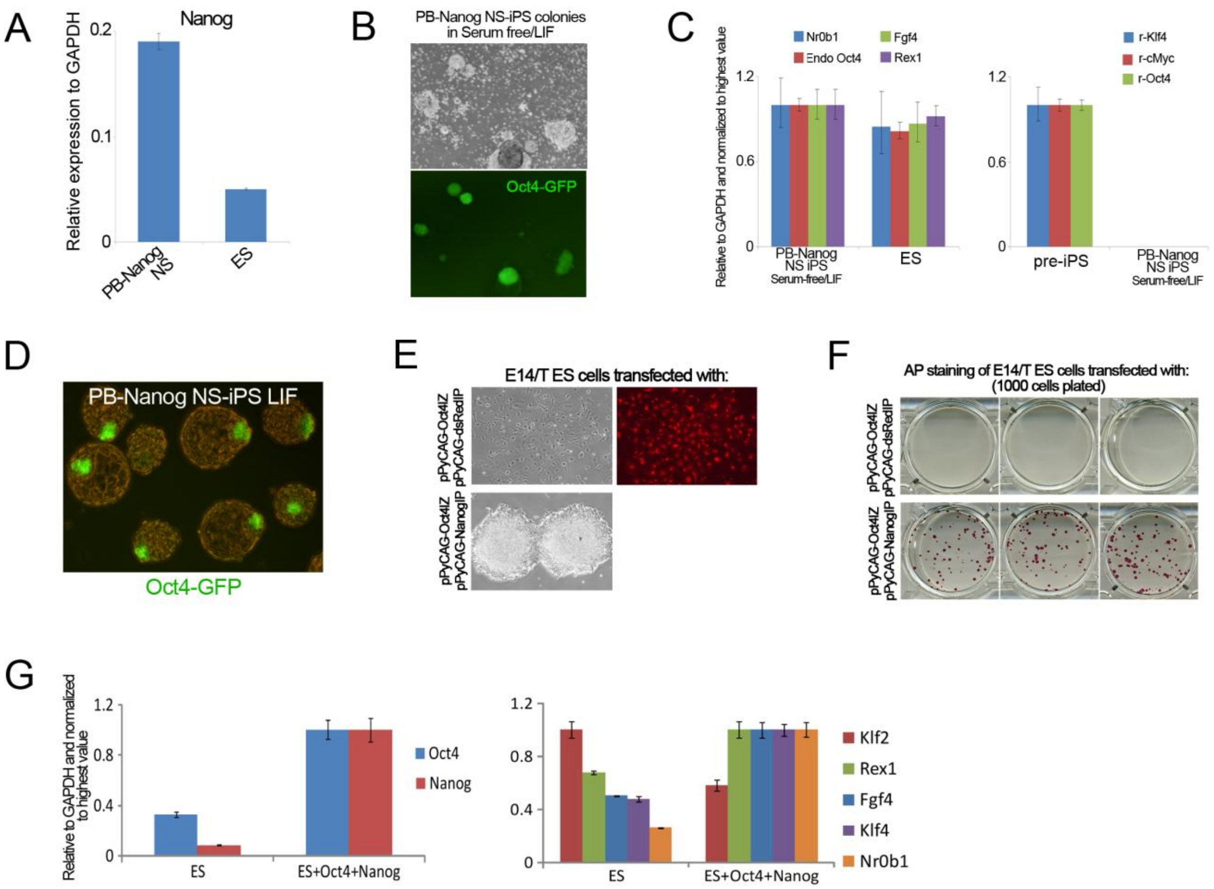

Figure S3. Nanog Mediates Reprogramming of NS Cells in Serum-Free Medium with LIF and Counteracts Differentiation Induced by High Oct4 in ES Cells (Related to Figure 3)
(A) qRT-PCR analysis of Nanog transcript expression in adult NS cells stably transfected with PB-Nanog. Error bars indicate one SD.
(B) Oct4-GFP images of nascent iPS colonies emerging in serum-free medium with LIF 12 days after retroviral infection of PB-Nanog NS cells.
(C) qRT-PCR analysis of endogenous pluripotency genes (*Left*) and of retroviral transgenes (*Right)* in iPS cells derived from PB-Nanog NS cells in serum-free medium with LIF. Error bars indicate the range of fold change relative to the sample with highest expression.
(D) Oct4-GFP reporter activity in blastocysts 52h after morula aggregation of PB-Nanog iPS cells derived from NS cells in serum-free medium with LIF.
(E) Morphology of ES cells selected for stable expression of episomal vectors expressing Oct4 and dsRed or Oct4 and Nanog and plated at clonal density under continued selection.
(F) Alkaline phosphatase staining after clonal density plating of ES cells overexpressing Oct4 or co-expressing Oct4 and Nanog.
(G) qRT-PCR analysis of pluripotency gene expression in ES cells co-expressing Oct4 and Nanog. Error bars indicate the range of fold change relative to the sample with highest expression.

Supplemental Experimental Procedures

Cell Culture

ES cells and pre-iPS cells were cultured in GMEM containing 10% FCS, 1x NEAA, 1 mM sodium pyruvate, 0.1 mM 2-mercaptoethanol, 2mM L-glutamine, supplemented with LIF (complete medium). Where indicated 3 μM 5-aza-cytidine (Sigma) was added to complete ES cell medium. STO fibroblasts or DsRed-expressing fibroblasts treated with mitomycin-C were used as feeder layer for the expansion of pre-iPS cells. 2i-iPS cells were generated and maintained without feeders in N2B27 (Stem Cell Sciences, catalogue number: SCS-SF-NB-02) supplemented with LIF and 2i inhibitors (Ying et al., 2008), CHIR99021 (3μM) and PD0325901 (1μM) obtained from the Division of Signal Transduction Therapy, University of Dundee. NS cells were maintained in NDiff basal RHB-A (Stem Cell Sciences, catalogue number: SCS-SF-NB-01) supplemented with 10 ng/ml of both EGF and FGF-2. EpiSCs derived from E5.5 Oct4GiP epiblast were cultured in activin A (20 ng/ml) and Fgf2 (12 ng/ml) in N2B27 medium. EpiSC were transfected using LipofectamineTM 2000 (Invitrogen) with 1μg of PB-CAG-DsRed-ires-Hygro-CAG-Nanog plus 2 μg PBase expression vector, pCAGPBase to obtain cells that express constitutively Nanog and hygromycin resistance. Where indicated, puromycin (1 μg/ml) selection for Oct4 reporter activity was applied.

Retroviral Infection

Retroviral infection was performed as described [2-3] with minor modifications. We infected mouse embryonic fibroblasts (MEFs) with retroviral transgenes expressing Oct4, Klf4, c-Myc and Sox2 (OKMS). We infected neural stem (NS) cells with retroviral transgenes expressing Oct4, Klf4 and c-Myc (OKM), omitting Sox2 as this factor was found to be dispensable for NS cell reprogramming [4-5]. Plat-E cells were seeded at 2×10^6 cells per 100-mm dish. The following day, 9 μg of pMX-based retroviral vectors were introduced separately into Plat-E cultures using 27 μl of FuGENE 6 transfection reagent. After 24h, the medium was replaced with 10 ml of DMEM containing 10% FCS. Target cells (MEFs and NS cells) were seeded at 1.2 x 10^5 cells per 35-mm dish coated with gelatin. The following day, virus-containing supernatants from Plat-E cultures were filtered through a 0.45-μm cellulose acetate filter. Equal volumes of the supernatants were mixed and supplemented with polybrene at the final concentration of 4μg ml^{-1}. Cells were incubated in the virus/polybrene-containing supernatants for 24 h. MEF infections were switched to ES cell culture conditions at this time point, whereas NS cell infections were restored to NS cell culture medium. Three days after transduction, NS cell infections were changed into ES cell medium. For further expansion pre-iPS cells were replated onto feeders at day 5 in medium containing serum and LIF. To establish clonal lines of pre-iPS cells individual colonies were picked and expanded 10-14 days after infection. pMXs-gw plasmids; pMXs-Oct4, pMXs-Klf4, pMXs-cMycT58 and pMXs-Sox2 were obtained from Addgene repository.

ES Cell Transfection with Episomal Oct4 and Nanog Expression Vectors

0.5×10^6 supertransfectable E14T ES cells were plated per well in a 6-well plate and lipofected the next day with 2 µg of episomal vectors expression vectors: pPyCAG-Oct4-ires-Zeo and pPyCAG-dsRed-ires-puro or pPyCAG-Oct4-ires-Zeo and pPyCAG-Nanog-ires-puro. Dual zeocin (200 µg/ml) and puromycin (1 µg/ml) selection was applied in ES cell medium for 10 days. At this point cells were plated at clonal density (1000 cells/well in a 6-well plate) under continued selection, and alkaline phophatase staining was performed 10 days later.

Blastocyst Injection and Morula Aggregation

Chimaeras were generated by standard microinjection methodology using host blastocysts of strain C57BL/6. Transmission from cultured cells manifests in agouti coat color. For morula aggregation, Oct4GiP reporter iPS cells or EpiSCs were combined with E2.5 MF1 morulae and maintained in culture for 52 hours. Cultured embryos were examined by fluorescence microscopy.

Western Blotting

The following antibodies and dilutions were used: mouse monoclonal antibodies to Oct4 (C-10) (1:500) and α-tubulin (1:5000) from Santa Cruz Biotechnology and rabbit polyclonal antibodies to phospho-Erk (1:1000) from Cell Signaling and total Erk (1:1000) from Cell Signaling. For quantitative Western analysis, Oct4 or α-tubulin immunoreactivity was detected with an anti-mouse infrared IRDye-labeled 800CW secondary antibody (1:15000) from LI-COR. Nitrocellulose membranes were analyzed with the Odyssey Near Infra Red Scanner. Intensity of Oct4 bands was normalized to α-tubulin from two gels for each experiment.

Immunofluorescent Staining

Cells were cultured overnight on glass slides and fixed directly in 4% PFA, followed by permeabilization in 0.5% Triton-X. The following antibodies and dilutions were used: mouse monoclonal antibody to Oct4 (1:100) from Santa Cruz Biotechnology (C-10, cat no: sc-5279), rabbit polyclonal antibody to trimethyl H3-K27 (1:500) from Upstate (cat no: 07-449) and mouse monoclonal antibody to Dnmt3b (1:500) from Imgenex.

Bisulfite Sequencing

Genomic DNA was extracted using the DNeasy Blood and Tissue Kit (Qiagen). Bisulfite treatment was performed using the CpGenome Fast DNA modification kit (Chemicon) or EpiTect Bisulfite Kit (Qiagen). See Primer Table for details of the primers used to amplify the *Nanog* promoter and *Oct4* distal enhancer and promoter regions. Amplified products were cloned into pCR2.1-TOPO (Invitrogen). 5-10 randomly selected clones were sequenced with the M13 forward and M13 reverse primers for each gene at the DNA sequencing facility of the Department of Biochemistry, University of Cambridge. Results were analyzed using

Quantification Tool for Methylation Analysis (QUMA, http://quma.cdb.riken.jp/).

Chromatin Immunoprecipitation

ChIP-IT Express (Active Motif) was used according to the supplier's recommendations. Cells were cross-linked using 1% formaldehyde for 10 min at room temperature. Formaldehyde was quenched by a 5 minutes incubation with glycine, cells were rinsed twice with cold PBS, collected by scraping and pelleted at 2500 rpms for 10 minutes at 4°C. Frozen pelleted cells were thawed and resuspended in lysis buffer, rotated for 30 minutes at 4°C, dounced and centrifuged at 5000 rpms for 10 mins at 4°C. Pelleted nuclei were resuspended in shearing buffer. Chromatin was then sonicated using a Bioruptor™ 200 (Diagenode), high frequency, 0.5 min/0.5min, for 10 minutes twice. Sonicated chromatin was analysed in 1% agarose gel, to confirm efficient sonication. Input was collected for further analysis. 5-15 µg of chromatin was incubated with 2µg of rabbit IgG (control, Abcam, ab6742) or rabbit anti-Nanog (Bethyl Laboratories, A300-397A) for 1 hour at 4°C and subsequently with protein G magnetic beads. After overnight immunoprecipitation at 4°C, beads were washed 3 times with ChIP buffer 1 and 2 times with ChIP buffer 2. After elution and reverse cross-link (95°C for 15 minutes), samples were treated with proteinase K for 1 hour. Purified DNA and 1% input were analysed by Taqman qPCR, using 4-fold dilutions of the concentrated input for standard curves and triplicates per sample. Occupancy is plotted as fold enrichment over IgG, after normalization to the input, and error bars represent standard deviation of the technical replicates of the qPCR for each experiment. Primers are listed in Table S1.

Imaging and Flow Cytometry

Slides were analyzed on a confocal microscope (Leica TCS SP5) and processed with Leica software and Adobe Photoshop. Images of live cells were captured with a Leica CTR microscope and processed with Leica software and Adobe Photoshop. Flow cytometry analyses were performed using a Dako Cytomation CyAn ADP high-performance cytometer with FlowJo and Summit software. Cell sorting was performed using a MoFlo high-speed cell sorter.

Quantitative Real Time PCR

Total RNA was extracted using the RNeasy kit (Qiagen), and cDNA generated using Superscript III (Invitrogen). Unless otherwise indicated, relative expression levels of Fgf4, Nr0b1, Nanog, Rex1, Oct4, Klf4, Klf2, Fgf5, Lefty and T (Brachyury) were determined using the TaqMan Fast Universal PCR Master Mix (Applied Biosystems) and FAM-labeled TaqMan gene expression assays. Relative expression levels of retroviral Oct4, Klf4, Sox2, c-Myc, and endogenous Oct4, were determined using Custom TaqMan Gene Expression Assays. Average threshold cycles were determined from triplicate reactions and the levels of gene expression were normalized to GAPDH (VIC-labeled endogenous control assay). Error bars indicate ±1 standard deviation or range of fold change relative to reference sample, as indicated in the legend. Relative expression levels of Dnmt3a and Dnmt3b were determined using gene-specific primers and the Fast SYBR Green Master Mix (Applied Biosystems). Mean quantity of expression was determined from triplicate reactions and a standard curve. Expression levels were normalized to GAPDH. Error

bars indicate range of fold change relative to the sample with highest expression. qRT-PCR experiments were performed on a 7900 HT Fast Real-Time PCR System or StepOnePlus Real Time PCR System (Applied Biosystems). Quantification of gene expression during inducible tOct4 induction in ES cells (Figure S1P) was performed using gene-specific primers and the Bio-Rad iQ SYBRgreen system. Relative expression to GAPDH was normalized to the 0h sample in two independent clones. See the Primer Table for details of primers and gene expression assays used in this study.

Table S1. Primers Used in This Study

GAPDH-F	CCCACTAACATCAAATGGGG	SYBR green real time PCR
GAPDH-R	CCTTCCACAATGCCAAAGTT	
Nanog-F	AAGATGCGGACTGTGTTCTC	SYBR green real time PCR
Nanog-R	CGCTTGCACTTCATCCTTTG	
Fgf4-F	GAGGCGTGGTGAGCATCTTC	SYBR green real time PCR
Fgf4-R	TCCGCCCGTTCTTACTGAGG	
Sox2-F	GGCGGCAACCAGAAGAACAG	SYBR green real time PCR
Sox2-R	GCTTGGCCTCGTCGATGAAC	
Gata6-F	TACACAAGCGACCACCTCAG	SYBR green real time PCR
Gata6-R	ATGTAGAGGCCGTCTTGACC	
Oct4-F	GGCGTTCTCTTTGGAAAGGTGTTC	SYBR green real time PCR
Oct4-R	CTCGAACCACATCCTTCTCT	
Brachyury-F	TGCTGCAGTCCCATGATAAC	SYBR green real time PCR
Brachyury-R	CAGACCAGAGACTGGGATAC	
Dnmt3a-F	GAGGGAACTGAGACCCCAC	SYBR green real time PCR
Dnmt3a-R	CTGGAAGGTGAGTCTTGGCA	
Dnmt3b-F	GTTAATGGGAACTTCAGTGACCA	SYBR green real time PCR
Dnmt3b-R	CTGCGTGTAATTCAGAAGGCT	
Retr. Oct4-F	TGGTACGGGAAATCACAAGTTTGTA	Custom TaqMan gene expression assay for retroviral transgene
Retr. Oct4-R	GGTGAGAAGGCGAAGTCTGAAG	
Retr. Oct4-probe	FAM-CACCTTCCCCATGGCTG-MGB	
Retr. Klf4-F	TGGTACGGGAAATCACAAGTTTGTA	Custom TaqMan gene expression assay for retroviral transgene
Retr. Klf4-R	GAGCAGAGCGTCGCTGA	
Retr. Klf4-probe	FAM-CCCCTTCACCATGGCTG-MGB	
Retr. cMyc-F	TGGTACGGGAAATCACAAGTTTGTA	Custom TaqMan gene expression assay for retroviral transgene
Retr. cMyc-R	GGTCATAGTTCCTGTTGGTGAAGTT	
Retr. Myc-probe	FAM-CCCTTCACCATGCCCC-MGB	
Retr. Sox2-F	TGGTACGGGAAATCACAAGTTTGTA	Custom TaqMan gene expression assay for retroviral transgene
Retr. Sox2-R	GCCCGGCGGCTTCA	
Retr. Sox2-probe	FAM-CTCCGTCTCCATCATGTTAT-MGB	
End. Oct4-F	TTCCACCAGGCCCCC	Custom TaqMan gene expression assay for endogenous transcript
End. Oct4-R	GGTGAGAAGGCGAAGTCTGAAG	
End. Oct4-probe	FAM-CCCACCTTCCCCATGGCT-MGB	
Fgf4	Applied Biosystems ID: Mm00438917_m1	TaqMan gene expression assay
Nr0b1	Applied Biosystems ID: Mm00431729_m1	TaqMan gene expression assay
Nanog	Applied Biosystems ID: Mm02384862_g1	TaqMan gene expression assay
Rex1	Applied Biosystems ID: Mm03053975_g1	TaqMan gene expression assay
Oct4 (total)	Applied Biosystems ID: Mm00658129_gH	TaqMan gene expression assay

Klf4 (total)	Applied Biosystems ID: Mm00516104_m1	TaqMan gene expression assay
Klf2	Applied Biosystems ID: Mm01244979_g1	TaqMan gene expression assay
Fgf5	Applied Biosystems ID: Mm00438919_m1	TaqMan gene expression assay
Lefty	Applied Biosystems ID: Mm00438615_m1	TaqMan gene expression assay
T (brachyury)	Applied Biosystems ID: Mm01318252_m1	TaqMan gene expression assay
GAPDH	Applied Biosystems ID: 4352339E	TaqMan gene expression assay
Nanog S	GATTTTGTAGGTGGGATTAATTGTGAATTT	Bisulfite methylation analysis of *Nanog* promoter
Nanog AS	ACCAAAAAAACCCACACTCATATCAATATA	
Oct4-DE F	GGTTTTAGAGGTTGGTTTTGGG	Bisulfite methylation analysis of *Oct4* distal enhancer
Oct4-DE R	CATCTCTCTAACCCTCTCCATAAATC	
Oct4-P F1	GGTTTTTTAGAGGATGGTTGAGTG	Round 1 primers for bisulfite methylation analysis of *Oct4* promoter
Oct4-P R1	TCCAACCCTACTAACCCATCACC	
Oct4-P F2	GGTTAGAGGTTAAGGTTAGAGGGTG	Round 2 (nested) primers for bisulfite methylation analysis of *Oct4* promoter
Oct4-P R2	CCCCCACCTAATAAAAATAAAAAAA	
ChIP CR4 F	GCATAACAAAGGTGCATGATAGCT	Taqman assay for ChIP analysis of mouse Oct4 distal enhancer (CR4 element)
ChIP CR4 R	AAATAAAGGCAGCGACTTGGAA	
ChIP CR4 probe	TAGCCCTTCCTTAATCTGCTA	
ChIP desert F	GATTGCAGAGTAAGATCCCTTGAT	Taqman assay for ChIP analysis of gene desert region on chromosome 5
ChIP desert R	GCGTAAGTTCTACATGCTGCTTTA	
ChIP desert probe	CACTTGAAAGAATGACAGTGCAATTCA	

Supplemental References

1. Silva, J., Nichols, J., Theunissen, T.W., Guo, G., van Oosten, A.L., Barrandon, O., Wray, J., Yamanaka, S., Chambers, I., and Smith, A. (2009). Nanog is the gateway to the pluripotent ground state. Cell *138*, 722-737.
2. Takahashi, K., and Yamanaka, S. (2006). Induction of pluripotent stem cells from mouse embryonic and adult fibroblast cultures by defined factors. Cell *126*, 663-676.
3. Takahashi, K., Okita, K., Nakagawa, M., and Yamanaka, S. (2007). Induction of pluripotent stem cells from fibroblast cultures. Nat Protoc *2*, 3081-3089.
4. Kim, J.B., Zaehres, H., Wu, G., Gentile, L., Ko, K., Sebastiano, V., Arauzo-Bravo, M.J., Ruau, D., Han, D.W., Zenke, M., et al. (2008). Pluripotent stem cells induced from adult neural stem cells by reprogramming with two factors. Nature *454*, 646-650.
5. Silva, J., Barrandon, O., Nichols, J., Kawaguchi, J., Theunissen, T.W., and Smith, A. (2008). Promotion of reprogramming to ground state pluripotency by signal inhibition. PLoS Biol *6*, e253.
